# On Meta-Geometric Gravity, Emergent Holography and Naturalness

**DOI:** 10.3390/e28101095

**Published:** 2026-10-08

**Authors:** Andrea Addazi

**Affiliations:** 1 School of Physics and Astronomy, Anqing Normal University, Anqing 246133, China; addazi@scu.edu.cn; 2 Institute of Astronomy and Astrophysics, School of Mathematics and Physics, Anqing Normal University, Anqing 246133, China; 3 CAS Key Laboratory for Research in Galaxies and Cosmology, School of Astronomy and Space Science, University of Science and Technology of China, Hefei 230026, China; 4 Laboratori Nazionali di Frascati INFN, 00044 Frascati, Italy

**Keywords:** meta-geometric gravity, topological theories, emergent spacetime, gauge theories of gravity, cosmological constant, holographic principle

## Abstract

We show that a discrete quantization law for cosmological entropy emerges naturally within a meta-geometric gauge theory of gravity, when the standard de Sitter entropy relation is combined with the topological structure of the theory. Our framework is based on a pre-metric action containing a MacDowell–Mansouri term with a spontaneously broken (A)dS gauge symmetry reduced to the Lorentz subgroup. We derive an inverse proportionality between the topological θ-angle and the cosmological constant, which enforces a topological quantization condition. As a consequence, the de Sitter entropy becomes quantized and scales linearly with a topological quantum number. This quantization provides a natural entropic stabilizer and a quantum topological protection for a large cosmological constant, thereby offering a microscopic gauge-theoretic realization of the holographic naturalness conjecture. We also discuss possible emergence of the N-portrait in cosmology and a dynamical relaxation of the vacuum energy.

## 1. Introduction

In recent works [1,2,3,4,5,6,7,8,9], we have developed a pre-geometric framework in which General Relativity emerges from the spontaneous symmetry breaking of an (A)dS gauge group to the Lorentz group. This construction builds upon the MacDowell–Mansouri action [10] and subsequent developments [11,12], introducing a gauge-covariant potential [13]. A central feature of this approach is that both the Planck scale $M_{\mathrm{Pl}}$ and the cosmological constant Λ are emergent quantities, determined by the symmetry-breaking parameters of the underlying gauge theory [1] (See also Refs. [14,15,16,17,18,19,20,21] or other references on related subjects.).

More precisely, the model is based on the spontaneous breaking of SO(1,4) to SO(1,3), with the fundamental fields consisting of a gauge connection $A_{\mu }$ in the adjoint representation and a meta-Higgs scalar Φ in the fundamental representation (We use the term “meta-Higgs” to distinguish this field from the Standard Model Higgs boson.)—the precise internal index structure will be specified below. Crucially, in contrast to many other proposals in the literature, no background tetrad or metric tensor is introduced a priori. Instead, the tetrad and spin connection are originally unified as components of the gauge potential, and their splitting emerges dynamically only after the meta-Higgs field acquires a vacuum expectation value.

To avoid any possible confusion, we emphasize that the term “pre-geometric” (or “meta-geometric”) is used here in a specific and limited sense. By this, we mean that the theory is formulated without presupposing a metric, tetrad, volume form, or spin connection as fundamental structures on the spacetime manifold; these objects arise dynamically from the fields Φ and $A_{\mu }$ upon spontaneous symmetry breaking. We do, however, assume a bare four-dimensional differentiable manifold, along with spacetime indices, derivatives, and a gauge connection. Thus, “pre-geometric” should be more accurately understood as *pre-metric* or *pre-tetradic*, and should not be interpreted as suggesting that no geometric structure whatsoever exists prior to symmetry breaking.

### 1.1. Entropy, Holography and Cosmological Constant Problem

It is well known that a semiclassical approach to quantum gravity, based on quantizing the metric field of General Relativity with a Euclidean Einstein–Hilbert Lagrangian, leads to the emergence of the Bekenstein–Hawking entropy area law for black holes [22,23]. This semiclassical analysis indicates that black holes possess well-defined thermodynamic laws and a characteristic temperature. Moreover, following the Gibbons–Hawking–York boundary approach, such results can be extended to the entire Universe in the presence of a cosmological horizon, the simplest case being de Sitter spacetime [24,25]. In a related development, the Hartle–Hawking no-boundary proposal [26,27] suggested that the Universe may tunnel from “nothing” to a wave function corresponding to a specific value of the cosmological constant Λ.

These results laid the groundwork for the idea that, in a broad sense, our Universe can be viewed as a hologram—the so-called holographic principle [28,29]—which later inspired the well-known AdS/CFT [30,31,32,33] and dS/CFT [34,35] conjectures. The latter is less established, being potentially affected by unstable modes, though this is not directly relevant to our present discussion, as we do not rely on conformal symmetry at the quantum level.

The fact that the entropy of de Sitter spacetime—or of quasi-de Sitter deformations thereof with a cosmological horizon—scales as the Hubble area immediately implies that it scales with the hierarchy $M_{\mathrm{Pl}}^{2}/\Lambda$. This observation suggests that a proper understanding of cosmological entropy may be the key to a novel reinterpretation of the cosmological constant hierarchy problem. Indeed, entropy in standard statistical mechanics is understood, from the microscopic perspective of Boltzmann’s atomic theory, as a count of degrees of freedom, i.e., as the logarithm of the number of accessible configurations. The crucial difference for black holes and de Sitter space is that the entropy scales with the area, implying that the information concerning the microscopic degrees of freedom is stored on the boundary rather than in the bulk.

This suggests that the cosmological constant may be more akin to a “temperature in vacuo” than to a zero-point energy—the latter perspective being the origin of the paradox articulated by Weinberg in his effective field theory approach to the problem [36]. From the perspective of spacetime thermodynamics, a destabilization of the cosmological constant from its observed value to a scale of order $M_{\mathrm{UV}}^{2}$ appears unreasonable for several reasons: (i) it would constitute a spontaneous violation of the second law of thermodynamics, as the de Sitter entropy would decrease from an enormous value of order $10^{120}$ to ∼1; (ii) it would correspond to a spontaneous reduction of the configuration space—analogous to a gas at room temperature spontaneously localizing in a corner while increasing its temperature rather than expanding—a process that is exponentially suppressed as $e^{-S_{dS}}$; and (iii) if entropy is interpreted as a count of microscopic degrees of freedom, such a process may destroy quantum information, violating unitarity.

These considerations inspired the holographic naturalness (HN) program [37,38,39,40]. In the HN framework, the cosmological constant problem is reinterpreted as originating from the ignorance of, and inaccessibility to, the whole information and entropy holographically stored in the vacuum. From the perspective of effective quantum field theory, particle physicists have traditionally assumed that the information in the vacuum is zero. This contrasts sharply with the holographic principle, which suggests instead that the amount of entropy is immense—$10^{120}$, precisely the hierarchy between the squared Planck energy and the cosmological constant. Any quantum radiative corrections from Standard Model fields, usually interpreted as polynomially divergent, cannot destroy entropy and information in the Universe; they are therefore probabilistically suppressed by a large exponential factor.

The same principle can be extended to the Higgs sector, though in a more subtle manner, leading to the introduction of the concept of “hairons” [37,38,39,40]. In an effective field theory approach, hairons were introduced as “spurion” or Stückelberg-like fields that carry quantum numbers (or quantum “hairs”), each contributing to the entropy. The idea that entropy counts quantum hairs was proposed [41,42], in order to circumvent the classical no-hair theorem [43,44,45,46,47,48], which states that classical black holes are characterized solely by their mass, electric charge, and angular momentum. The idea that such quantum hairs can be treated as background fields appears therefore reasonable. This perspective helps to eliminate dangerous ultraviolet divergences from bubble diagrams or scalar fields such as the Standard Model Higgs, within an effective Feynman diagrammatic and background field approach [37]. In addition, recent results propose to relate hairons to moduli spaces of gravitational multi-instantons, such as $S_{2}\times \cdots \times S_{2}/Z_{N}$ or instantons locally of the form $C_{2}/Z_{N}$ [40]. Nevertheless, such arguments rely on an effective field theory approach and semiclassical quantum gravity (or alternatively, coherent states), and may not be entirely satisfactory. The hairons are introduced as soft modes rather than heavy states, with Compton wavelengths as large as the Hubble radius; however, their microscopic origin remains obscure, and they may descend from the low-energy limit of a gauge theory of gravity or from some other ultraviolet completion.

### 1.2. Emergent Gravity and the Topological Multi-Vacua

As mentioned above, from the perspective of a meta-geometric gauge theory, the spacetime metric emerges only after spontaneous symmetry breaking, and consequently, so does any boundary term with an induced metric. Thus, holography itself is not fundamental in this framework but rather emerges as a derived concept. Accordingly, the entropy area law and the associated quantum information in cosmology are expected to be emergent phenomena, manifest only after the symmetry-breaking mechanism has occurred.

Inspired by this perspective, in the present work we elaborate on the cosmological entropy and the cosmological constant problem, focusing on emergent de Sitter spacetime. In particular, we consider the simple case of MacDowell–Mansouri gravity augmented by a topological θ-term and a meta-Higgs potential. We demonstrate that an entropy scaling linearly with a discrete quantum number naturally arises from this approach. This quantum number emerges from the topological sectors associated with the θ-angle and the Gauss–Bonnet sector, which in this model are predicted to be proportional to one another through the Immirzi parameter [49] related to an emergent Holst term [50].

A significant prediction of the model is that the Higgs vacuum expectation value is proportional to these topological angles and is therefore also quantum-mechanically discretized, reminiscent in a broad sense of axion physics. This implies a multi-vacuum structure for the meta-Higgs potential, with vacuum expectation values scaling linearly with the quantum number *k*. As a consequence, the vacuum energy density and the cosmological constant are also quantized in terms of *k*; each *k*-sector possesses a distinct cosmological constant and de Sitter entropy value. This leads to a reinterpretation of the de Sitter entropy as a topological charge of the vacuum. From the hairon perspective, it appears a duality of their counting number and such a topological quantum index *k*. We will see that this also corresponds to a symmetry degeneracy of the tetrad numbers and, dually, to the graviton numbers.

Moreover, the meta-geometric Higgs barriers separating the various vacua naturally can stabilize the cosmological constant through WKB suppressions that prohibit large excursions of the meta-Higgs field. For example, a transition from the observed cosmological constant to a value proportional to the squared Planck energy is exponentially suppressed. We will further comment on how a relaxation of the cosmological constant toward smaller values is entropically favored over the reverse process, thereby creating an asymmetry in the time evolution of the system.

## 2. Meta-Geometric Theory

### 2.1. Pre-Metric Action

We start with a gauge theory defined on a bare four-dimensional differentiable manifold, with neither a metric nor a tetrad structure assumed a priori. The gauge group is taken to be SO(1,4), with connection $A_{\mu }^{AB}$ and associated curvature $F_{\mu \nu }^{AB}$. General covariance is imposed throughout, and the sole internal structure is the tangent-space metric $\eta_{AB}=\mathrm{diag}\left(-,+,+,+,+\right)$. All action densities are constructed exclusively from the spacetime Levi-Civita symbol $\epsilon^{\mu \nu \rho \sigma }$, while an internal Levi-Civita symbol $\epsilon_{ABCDE}$ is likewise introduced for the gauge group. No background Minkowski metric, nor any associated tetrads, are presupposed. Internal indices are denoted by capital Latin letters A,B,C,…=0,1,2,3,4; Lorentz subgroup indices are indicated by lowercase Latin letters a,b,c,…=0,…,3; and spacetime indices are denoted by Greek letters μ,ν,ρ,σ=0,…,3.

The total action consists of three contributions:

(1) $$S_{1}+S_{2}+S_{3},$$

where $S_{1}$ denotes the MacDowell–Mansouri term [10],

(2) $$S_{1}=g_{\mathrm{MM}}\int d^{4}x\;\epsilon_{ABCDE}\;\epsilon^{\mu \nu \rho \sigma }F_{\mu \nu }^{AB}F_{\rho \sigma }^{CD}\Phi^{E},$$

with $\Phi^{A}$ being the Gravi-Higgs field in the fundamental representation of SO(1,4), and $g_{\mathrm{MM}}$ a coupling constant of mass dimension ${\left[\Phi \right]}^{-1}$.

The second contribution is the topological Pontryagin action:

(3) $$S_{2}=\frac{\theta }{32\pi^{2}}\int \mathrm{Tr}\left(F\wedge F\right),$$

where θ is an angular parameter. Note that this is a pre-metric Pontryagin term which will also contain the gravitational one after the spontaneous symmetry breaking.

The third term is given by

(4) $$S_{3}=\int |J|\;U(|\Phi |)\;d^{4}x,$$

with

(5) $$J=\epsilon_{ABCDE}\;\epsilon^{\mu \nu \rho \sigma }\;\nabla_{\mu }\Phi^{A}\;\nabla_{\nu }\Phi^{B}\;\nabla_{\rho }\Phi^{C}\;\nabla_{\sigma }\Phi^{D}\;\Phi^{E}$$

defining the pre-geometric gauge volume form, and $U\left(|\Phi |\right)\equiv U\left(\sqrt{|\eta_{AB}\Phi^{A}\Phi^{B}|}\right)$ denoting a potential that drives the scalar field toward its vacuum expectation value (VeV). Upon spontaneous symmetry breaking (SSB), the J-form reconstructs the covariant four-volume form. As we shall demonstrate, consistency of the theory requires U to be periodic, thereby giving rise to a countable infinity of vacuum sectors.

### 2.2. Spontaneously Broken Phase

Once the Higgs field acquires the VeV $\langle \Phi^{A}\rangle =v\delta_{5}^{A}$, the gauge field decomposes as

(6) $$A_{\mu }^{a5}=\epsilon e_{\mu }^{a},\;A_{\mu }^{ab}=\omega_{\mu }^{ab},$$

where $e_{\mu }^{a}$ denote the tetrads, $\omega_{\mu }^{ab}$ the spin connection (with a,b=0,…,3), and ϵ is a scale introduced to ensure dimensional consistency.

In the broken phase [1], the MacDowell–Mansouri action reduces to

(7) $$S_{1}\overset{\mathrm{SSB}}{\to }\int d^{4}x\;e\left(\frac{M_{\mathrm{Pl}}^{2}}{16\pi }R-\frac{M_{\mathrm{Pl}}^{2}}{8\pi }\Lambda +\alpha_{\mathrm{GB}}G\right),$$

with $e=\det e_{\mu }^{a}$, *R* the Ricci scalar, and G the Gauss–Bonnet density. The parameters $\left\{g_{\mathrm{MM}},v,\epsilon \right\}$ are mapped to $\left\{M_{\mathrm{Pl}},\Lambda ,\alpha_{GB}\right\}$ via

(8) $$M_{\mathrm{Pl}}^{2}=32g_{\mathrm{MM}}v\epsilon^{2},\;\Lambda =3\epsilon^{2},\;\alpha_{\mathrm{GB}}=-4g_{\mathrm{MM}}v.$$

Consequently, the Gauss–Bonnet coupling is related to the other parameters by

(9) $$\alpha_{GB}=-\frac{3M_{\mathrm{Pl}}^{2}}{8\Lambda },$$

a relation that is a pure prediction of the MacDowell–Mansouri framework and is absent in standard General Relativity.

Upon SSB, the topological θ-term generates the Holst term [50]:

(10) $$S_{II}⊃\frac{1}{\gamma }\int \epsilon^{\mu \nu \rho \sigma }R_{\mu \nu }^{ab}e_{\rho a}e_{\sigma b}\;d^{4}x,$$

where the Immirzi parameter [49] is given by

(11) $$\frac{1}{\gamma }=\frac{\theta \Lambda }{12\pi^{2}M_{\mathrm{Pl}}^{2}}=\frac{\theta }{|\theta_{GB}|},$$

with $|\theta_{GB}|=32\pi^{2}\left|\alpha_{GB}\right|$.

In contrast to loop quantum gravity, where γ is a free quantization ambiguity [51], here it is uniquely fixed by the ratio of topological θ-angles. This unification of the Immirzi and θ ambiguities constitutes a distinctive feature of our construction.

## 3. Emergent Energy Levels from Spontaneous Symmetry Breaking

Equation (11) reveals a non-trivial interdependence among the topological angles, the cosmological constant, and the Higgs vacuum expectation value—an observation that underpins the subsequent discussion. In analogy with Yang–Mills theory, the gravitational θ-angle is periodic, with θ→θ+2π leaving the path integral invariant. The same periodicity applies to $\theta_{GB}\equiv 32\pi^{2}\alpha_{GB}$ in four dimensions, since the Gauss–Bonnet term is a topological invariant. The unified periodicity relation is given by

(12) $$\theta_{GB}^{\left(k\right)}=\gamma \left|\theta^{\left(k\right)}\right|=\theta_{GB}^{\left(0\right)}+2\pi k,\;k\in Z.$$

It is important to clarify why the shift θ→θ+2πk generates physically inequivalent vacuum sectors rather than a mere relabeling. In ordinary Yang–Mills theory, the θ-angle is an arbitrary angle parameter. Here, however, the situation is qualitatively different because θ is not an external parameter but is dynamically related to the vacuum expectation value of the meta-Higgs field through the Immirzi parameter relation, Equation (11). Specifically, the periodicity of the gravitational θ-angle is tied to the periodicity of the meta-Higgs potential, Equation (16), which exhibits an infinite tower of degenerate minima separated by barriers. Each minimum corresponds to a distinct value of ${\langle \Phi \rangle }^{\left(k\right)}∼k$, and through Equations (8) and (11), to a distinct value of $\Lambda^{\left(k\right)}$. Thus, the *k*-sectors are not related by a simple shift of an external parameter; they are separated by finite-action barriers and represent genuinely distinct vacuum states of the same theory. The degeneracy is broken by the potential, and transitions between sectors require quantum tunneling.

Furthermore, the quantization condition $\theta_{GB}^{\left(k\right)}=\theta_{GB}^{\left(0\right)}+2\pi k$ arises from the requirement that the partition function be invariant under large gauge transformations. Since $\theta_{GB}$ is related to Λ through Equation (11), the periodicity of the topological angle directly induces a discrete spectrum for the cosmological constant. This is not an assumption but a consequence of the topological structure of the theory combined with the dynamical relation between θ, Λ, and *v*.

Inserting this into Equation (11) yields the quantized cosmological constant

(13) $$\Lambda^{\left(k\right)}=\frac{12\pi^{2}M_{\mathrm{Pl}}^{2}}{\gamma (|\theta^{\left(0\right)}|+2\pi k)},$$

with the associated vacuum energy spectrum

(14) $$\epsilon^{\left(k\right)}=\frac{2\pi M_{\mathrm{Pl}}}{\sqrt{\gamma (|\theta^{\left(0\right)}|+2\pi k)}}.$$

We now discuss the allowed range of the topological index *k*. Although the periodicity relation is formally defined for k∈Z, the physical spectrum is restricted to k≥0. There are three reasons for this. First, the de Sitter entropy $S_{\mathrm{dS}}^{\left(k\right)}=\frac{\gamma }{4\pi }\left(\right|\theta^{\left(0\right)}\left|+2\pi k\right)$ must be non-negative; for *k* negative and sufficiently large in magnitude, it would become negative. Second, the effective cosmological constant $\Lambda^{\left(k\right)}\propto 1/\left(\theta^{\left(0\right)}+2\pi k\right)$ must be positive for an expanding de Sitter universe; for $\theta^{\left(0\right)}\in \left(0,2\pi \right]$ this is automatically satisfied for all k≥0, whereas negative *k* can drive $\Lambda^{\left(k\right)}$ through zero and change its sign. Third, the relaxation dynamics derived in Section 5 proceeds by a monotonic drift toward larger *k*, starting from an initial positive value; restricting the physical spectrum to k≥0 is therefore natural in the relaxation picture (The special value $\theta^{\left(0\right)}+2\pi k=0$, if it were realized for an integer *k*, would correspond to a singularity of the effective description, with Λ→∞ and $S_{\mathrm{dS}}\to 0$. This is a boundary of the effective field theory regime, not a physical state; in the large-*k* regime relevant for the observed Universe ($k∼10^{120}$), the denominator is manifestly positive and large, and the entire discussion is unambiguous).

The k=0 sector corresponds to $\epsilon^{\left(0\right)}=2\pi M_{\mathrm{Pl}}/\sqrt{\gamma |\theta^{\left(0\right)}|}$, which is of order of the Planck scale for $\theta^{\left(0\right)}\in \left(0,2\pi \right]$. In the large-*k* regime, $k∼10^{120}$, the observed value of the cosmological constant is recovered, with the asymptotic scalings $\epsilon^{\left(k\right)}∼M_{\mathrm{Pl}}/\sqrt{k}$ and $\Lambda^{\left(k\right)}∼M_{\mathrm{Pl}}^{2}/k$. Newton’s constant, and hence the Planck mass $M_{\mathrm{Pl}}$, remain independent of *k*.

The periodicity of θ also induces a monodromy in the Higgs potential, giving rise to a discrete infinity of vacuum expectation value configurations:

(15) $${\langle \Phi \rangle }^{\left(k\right)}=v^{\left(k\right)}∼\left|\theta^{\left(k\right)}\right|∼k,$$

in Planck units. The Higgs VeV is therefore labeled by the same integer *k* that characterizes the vacuum sector.

A potential that realizes this periodic VeV structure is

(16) $$V=h_{\mathrm{SSB}}\left|J\right|{\left[1-\cos\left(128\pi^{2}g_{\mathrm{MM}}\left(|\Phi |-v\right)\right)\right]}^{\xi },$$

where $h_{\mathrm{SSB}}$ sets the overall scale, $g_{\mathrm{MM}}$ is the MacDowell–Mansouri coupling, and ξ≥0 is a power-law exponent. This axion-like potential exhibits multiple minima separated by large barriers, thereby stabilizing the different vacuum sectors against large field excursions—in following discussions we will consider the simplest case ξ=1.

## 4. Holographic Naturalness

The holographic principle provides profound insights into the nature of dark energy through the entropy of de Sitter spacetime:

(17) $$S_{\mathrm{dS}}∼\frac{A_{H}}{A_{P}}∼\frac{M_{P}^{2}}{\Lambda }∼N,$$

where $A_{H}$ is the Hubble horizon area and *N* represents the number of holographic qubits stored in spacetime. This relation reveals several fundamental aspects. First, Equation (17) implies that the vacuum information content, which is of order $10^{120}$ for the observed cosmological constant, vastly exceeds that of baryonic matter and radiation, for which the cosmic microwave background entropy is of order $10^{88}$. Second, the cosmological scale $\sqrt{\Lambda }$ corresponds to the Gibbons–Hawking temperature $T_{\mathrm{dS}}$. Third, the hierarchy $\Lambda /M_{P}^{2}∼S_{\mathrm{dS}}^{-1}$ relates directly to the inverse entropy. In this light, the equation points toward a profound shift in understanding, recasting the cosmological constant problem in terms of entropy and information.

The stability of the observed low-energy vacuum against quantum decay to high-energy states is a central problem in cosmology. Within the holographic naturalness framework, this stability is not a result of fine-tuning but a natural consequence of entropic suppression [37]. Quantum transitions from the observed cosmological constant state to high-energy vacuum states are exponentially suppressed by immense entropic barriers:

(18) $$\left|\Lambda \rangle \to \right|M_{P}\rangle \;\mathrm{or}\;|N\rangle \to |1\rangle ,$$

where the latter notation represents a transition from a state with a large number of holographic degrees of freedom, corresponding to the observed Universe, to one with far fewer, corresponding to a high-energy vacuum. The corresponding transition amplitudes are

(19) $$\langle \Lambda |M_{P}\rangle ∼e^{-S_{\mathrm{dS}}/2}\ll 1,\;\langle N|1\rangle ∼e^{-N/2}≃e^{-10^{120}/2}.$$

This suppression is not merely analogous to but fundamentally mirrors non-perturbative effects in gauge theories and gravity, such as gravitational instanton tunneling:

(20) $$e^{-S_{E}}∼e^{-1/\alpha_{G}\left(\Lambda \right)},\;\alpha_{G}\left(\Lambda \right)=\Lambda /M_{P}^{2}∼S_{\mathrm{dS}}^{-1}∼N^{-1}.$$

Here, the inverse coupling strength $1/\alpha_{G}\left(\Lambda \right)$ plays the role of the action, which is set by the de Sitter entropy itself. This connection reveals that the suppression is non-perturbative in nature and cannot be seen at any finite order in perturbation theory.

Several key considerations follow from this principle. From an information-theoretic perspective, the suppression factor $e^{-N}$ reveals that the cosmological constant problem is fundamentally a question of statistical typicality. The Hilbert space of quantum gravity is vast, with a dimension set by the horizon entropy: $\dim\left(H\right)∼e^{S_{\mathrm{dS}}}=e^{N}$. Within this space, the states that resemble our observed low-energy de Sitter vacuum are overwhelmingly numerous; they form a set of measure 1 for a sufficiently coarse-grained observer. A transition to a high-energy vacuum $|M_{P}\rangle$ represents a leap into an exponentially small, atypical corner of the Hilbert space. The suppression factor $e^{-N}$ is precisely the measure of this atypicality. The air-in-a-corner analogy illustrates that what we perceive as a law—that gas fills its container and that Λ is small—is ultimately a statistical effect. We do not observe a large cosmological constant for the same reason we do not observe shattered cups spontaneously reassembling: such processes are not impossible, but their probability is exponentially small in the number of underlying degrees of freedom.

The continuous interaction between the vacuum state and the vast hairon environment leads to rapid decoherence. This environmentally-induced superselection, or einselection, effectively pins the system to its current state, making a coherent quantum jump to a different vacuum state dynamically forbidden over cosmological timescales. This can be viewed as a cosmological-scale manifestation of the quantum Zeno effect [52,53]. The Universe is freezing the vacuum into its current low-energy state. This Zeno-like stabilization is more fundamental than a simple potential barrier; it is a dynamical, information-theoretic mechanism that explains why the vacuum does not explore the vast landscape of possible high-energy states, rendering the observed, large-entropy cosmological constant state a stable attractor.

This mechanism directly addresses Weinberg’s no-go theorem, which is grounded on the principle of locality and assumes that the effective cosmological constant can vary continuously. In the holographic naturalness framework, however, the superselection of the state by the environment means that the value of Λ is not a free parameter subject to adjustment; it is a stable, "frozen" property of the selected quantum state. The holographic principle is inherently non-local: the state of the interior bulk, corresponding to our observable Universe, is encoded on the boundary, namely the cosmological horizon—see, for example, Refs. [54,55,56,57,58,59,60] discussing non-local aspects of holography from different perspectives. This implies several important consequences. First, the value of Λ is not a local degree of freedom that can be adjusted point-by-point like a scalar field; rather, it is a global property of the entire cosmological quantum state, determined by the number of holographic degrees of freedom *N* on the horizon. The physics is therefore governed not by a local Λ but by the global entropy $S_{\mathrm{dS}}∼N∼M_{P}^{2}/\Lambda$. In the holographic naturalness framework, the vast hairon environment on the horizon performs a continuous, non-local “measurement” of the cosmological state, leading to environment-induced superselection: the Hilbert space of possible vacuum states is split into superselection sectors, each corresponding to a different value of *N* and thus Λ; a transition from one sector to another, such as |N〉→|1〉, is not a continuous, local adjustment but rather a global, non-perturbative leap that requires changing the number of fundamental holographic qubits; and the probability for such a non-local transition is exponentially suppressed by the entropy difference.

A sharp contrast emerges when comparing this framework with standard quantum field theory. Standard QFT computations, which quantize fields on a fixed background while ignoring the dynamical and information-carrying properties of the gravitational Hilbert space, provide an incomplete description of vacuum energy. Physicists treat the vacuum as a system with few degrees of freedom, missing the crucial entropic stabilization that arises from the holographic nature of gravity. The chasm between the predictions of standard QFT and the observed value of the cosmological constant finds its origin in a fundamental conceptual mismatch: QFT provides a powerful but inherently local and non-gravitational description of matter, which becomes profoundly inadequate when confronted with the global, information-theoretic nature of gravity. Standard QFT computations are performed by quantizing field fluctuations on a fixed, inert background spacetime, typically Minkowski space. This framework is built on two core assumptions that fail in the presence of a dynamical cosmological horizon. First, the background is treated as passive and infinite: in QFT, the spacetime geometry itself is non-dynamical. The calculation of vacuum energy involves summing zero-point energies up to a Planck-scale cutoff, yielding a result of order $M_{P}^{4}$. The framework is therefore blind to the gravitational back-reaction of its own calculation; it computes a source for gravity while ignoring how that source would instantly alter the gravitational field itself. Second, the vacuum is treated as a simple, low-entropy state: within the Fock space of QFT, the vacuum |0〉 is the unique state annihilated by all lowering operators. It is treated as a simple, elementary state with essentially zero entropy and few relevant degrees of freedom. This perspective leads to the notion that the vacuum can freely tunnel or fluctuate into other states, as the phase space for such transitions appears unconstrained. The vacuum energy is thus viewed as a property of this simple state, susceptible to large quantum corrections from virtual particle loops.

The holographic naturalness framework reveals that this picture is not just slightly off but fundamentally incorrect when gravity is involved. Gravity holographically encodes the information contained within a spatial volume onto its bounding surface. A universe with a cosmological constant possesses a cosmological horizon, and the number of fundamental, irreducible degrees of freedom associated with this spacetime is finite and determined by the horizon area. This leads to a radically different picture. The observed vacuum state |Λ〉 is not simple but it is obtained from N soft modes constituting the de Sitter macrostate. It is a collective state of the holographic degrees of freedom, or hairons, on the horizon, with an entropy $S_{\mathrm{dS}}∼10^{120}$. It is as complex and structured as a macroscopic thermodynamic system in equilibrium. The stability of Λ is therefore stabilized statistically rather than by energetic minimization. A transition to a high-energy vacuum with a small horizon, such as |N〉→|1〉, would represent a catastrophic reduction in entropy, ΔS∼−N. In quantum statistical mechanics, the amplitude for such a process is exponentially suppressed, $∼e^{-N}∼e^{-10^{120}}$. This is the entropic stabilization—a thermodynamic effect completely invisible to standard QFT, which lacks the concept of gravitational entropy. Thus, the standard QFT computation of the vacuum energy is not merely a calculation that gives a large number; it is a calculation performed in the wrong physical paradigm. It mistakes a high-entropy, thermodynamically stable gravitational state for a low-entropy, fragile quantum state. It is akin to calculating the properties of a single water molecule and using it to predict the behavior of a tsunami, entirely missing the collective, thermodynamic laws that actually govern the system. The problem of the cosmological constant is therefore an artifact of applying a locally valid theory to a global, holographic context where its foundational assumptions break down.

### The Hairon Framework

Hairons, denoted by $\varphi_{h}\left(x,t\right)$, represent the quantum hairs [37] on the de Sitter horizon, providing a concrete field-theoretic realization of the holographic degrees of freedom. These are not arbitrary new fields but are emergent from the non-trivial, long-range quantum gravitational structure of the spacetime horizon. The vast number of hairons, $N∼S_{\mathrm{dS}}$, can undergo Bose–Einstein condensation, forming a coherent quantum state that permeates the horizon. This condensate is characterized by the de Sitter temperature $T_{\mathrm{dS}}=H/2\pi$, which sets the average energy per hairon: $\langle E_{h}\rangle =T_{\mathrm{dS}}∼\sqrt{\Lambda }$. This connects the microscopic hairon energy directly to the macroscopic cosmological scale.

The individual hairon excitations possess a tiny mass gap, $\Delta ∼\sqrt{\Lambda }/N$, which is inversely proportional to the total number of degrees of freedom. This is characteristic of systems with a large-*N* collective structure. While individual hairons are extremely soft, their collective effect is significant. The total energy scale of the condensate, given by the expectation value $N\Delta ∼M_{P}/\sqrt{N}$, represents a hierarchy between the Planck scale and the cosmological scale. This is not a fine-tuning but a direct consequence of the holographic scaling $N∼M_{P}^{2}/\Lambda$. In hairon systems, information scales as I≡ℏ/(RΔ)∼N, where $R∼\sqrt{N}\ell_{P}$, reconstructing the de Sitter entropy scaling [37,38,39].

Within the framework of effective field theory, hairons interact weakly with Standard Model fields. Their primary role is to modify the quantum amplitudes for processes that would otherwise destabilize the vacuum. In quantum field theory, the energy of empty space, or vacuum energy, is calculated using specific types of Feynman diagrams known as bubble diagrams. When a thermal background of hairons is introduced, the mathematical terms representing their thermal properties, or thermal insertions, are added to these diagrams. The effect of these terms is to impose an exponential suppression on the contributions from energy scales higher than the temperature of the hairon system, meaning that the calculation is effectively cut off at high energies. This provides a heuristic effective field theory picture of how the holographic degrees of freedom may stabilize the vacuum. A rigorous derivation of the non-perturbative suppression factor from the pre-geometric theory remains an open problem.

## 5. Quantization of de Sitter Entropy

Upon spontaneous symmetry breaking, the Gibbons–Hawking–York boundary term emerges from the Einstein–Hilbert action on the de Sitter horizon, yielding the standard de Sitter entropy:

(21) $$S_{\mathrm{dS}}=\frac{3\pi M_{\mathrm{Pl}}^{2}}{\Lambda }=\frac{A_{\mathrm{dS}}}{4G},$$

where the last equality invokes the Bekenstein–Hawking area law, with $A_{\mathrm{dS}}=12\pi /\Lambda$ denoting the horizon area.

It is important to state clearly what is and what is not derived from first principles in this work. We do not derive the Bekenstein–Hawking area law from the microscopic degrees of freedom of the pre-geometric theory; rather, we assume the standard semiclassical relation $S_{\mathrm{dS}}=A/\left(4G\right)$ and show that, within the meta-geometric framework, this relation implies a discrete spectrum for the entropy and the cosmological constant. The area law itself is treated as an emergent semiclassical result, consistent with the Gibbons–Hawking–York boundary term that arises upon spontaneous symmetry breaking. A fully microscopic derivation of the area law from the pre-geometric degrees of freedom remains an open problem and is beyond the scope of the present work.

Within the pre-geometric MacDowell–Mansouri framework, the de Sitter entropy admits several equivalent expressions in terms of the underlying parameters. Using Equation (8), we obtain

(22) $$S_{\mathrm{dS}}=32\pi \left|g_{\mathrm{MM}}\right|\;v.$$

This relation implies that the entropy vanishes in the limit v→0, i.e., when the gauge symmetry is restored. The VeV thus functions as an effective “entropy field” or quantum information-carrying order parameter, establishing a deep connection between cosmological entropy and the symmetry-breaking sector.

Furthermore, employing Equation (9) allows us to express the entropy in terms of the Gauss–Bonnet coupling. The starting point is the well-known result that, for a gravitational instanton with topology characterized by the Euler characteristic χ(M), the Gauss–Bonnet term contributes to the entropy with a coefficient proportional to χ(M). In the Euclidean path integral, the Gauss–Bonnet term is a topological invariant, and its contribution to the on-shell action is $S_{\mathrm{GB}}=-8\pi \alpha_{\mathrm{GB}}\chi \left(M\right)$. For a four-dimensional manifold with boundary, the Gauss–Bonnet term combines with the Gibbons–Hawking–York boundary term to give the correct de Sitter entropy. In our framework, the Gauss–Bonnet coupling $\alpha_{\mathrm{GB}}$ is not a subleading correction: it is directly tied to the leading contribution to the de Sitter entropy through the relation $\alpha_{\mathrm{GB}}=-3M_{\mathrm{Pl}}^{2}/\left(8\Lambda \right)$, which is a pure prediction of the MacDowell–Mansouri construction. This is in sharp contrast with standard General Relativity, where $\alpha_{\mathrm{GB}}$ is a free parameter, typically set to zero.

The key point is that in the sum over topologies (or, equivalently, over gravitational instanton configurations), the Gauss–Bonnet contribution to the entropy involves an average Euler characteristic 〈χ(M)〉. For orientable closed manifolds of genus *g*, the Euler characteristic is χ=2−2g, which becomes negative for g>2. When averaged over the relevant class of topologies, 〈χ(M)〉<0, so that $S_{\mathrm{dS}}=-8\pi \alpha_{\mathrm{GB}}\langle \chi \left(M\right)\rangle =8\pi |\alpha_{\mathrm{GB}}\left|\;|\langle \chi \left(M\right)\rangle \right|$. Absorbing the average Euler characteristic into the definition of the effective Gauss–Bonnet coupling, $|\alpha_{\mathrm{GB}}^{\mathrm{eff}}\left|\equiv \right|\alpha_{\mathrm{GB}}\left|\;|\langle \chi \left(M\right)\rangle \right|$, we obtain the relation $S_{\mathrm{dS}}=8\pi \left|\alpha_{\mathrm{GB}}^{\mathrm{eff}}\right|$ used in the manuscript. This is a novel prediction of the pre-geometric framework and distinguishes it from standard General Relativity, where the Gauss–Bonnet coupling does not control the leading de Sitter entropy:

(23) $$S_{\mathrm{dS}}=8\pi \left|\alpha_{\mathrm{GB}}\right|.$$

(For simplicity we omit |〈χ(M)〉| from now.) Combining this with Equation (11) yields a direct relation to the topological θ-angle:

(24) $$S_{\mathrm{dS}}=\frac{\gamma |\theta |}{4\pi }.$$

These expressions reveal that the de Sitter entropy is not merely a geometric quantity but is fundamentally rooted in the topological parameters of the gauge theory. Notably, the entropy depends on the Immirzi parameter γ, echoing analogous structures in loop quantum gravity [51]—a point to which we shall return below.

Comparing Equation (24) with the area law yields a relation between the de Sitter horizon area and the topological parameters:

(25) $$A_{\mathrm{dS}}=\frac{G}{\pi }\;\gamma \left|\theta \right|.$$

### 5.1. Topological Quantization of Entropy

The preceding relations directly imply a topological quantization of the de Sitter entropy. Substituting the quantized vacuum energy spectrum from Equation (14) into Equation (21), we obtain

(26) $$S_{\mathrm{dS}}^{\left(k\right)}=\frac{3\pi M_{\mathrm{Pl}}^{2}}{\Lambda^{\left(k\right)}}=\frac{\gamma }{4\pi }\left(|\theta^{\left(0\right)}|+2\pi k\right)$$

Thus, the entropy of the cosmological horizon takes discrete values indexed by the integer *k*, reflecting the underlying pre-geometric gauge structure.

The k=0 sector defines a minimal entropy unit:

(27) $$S_{\mathrm{dS}}^{\left(0\right)}=\frac{\gamma |\theta^{\left(0\right)}|}{4\pi }.$$

For illustrative values $\theta^{\left(0\right)}=0,\pi ,2\pi$, this yields $S_{\mathrm{dS}}^{\left(0\right)}=0,\gamma /4,\gamma /2$, respectively. The incremental contribution from higher *k*-levels is simply

(28) $$\Delta S_{\mathrm{dS}}^{\left(k\right)}=\frac{\gamma k}{2}.$$

The corresponding fundamental area cell is

(29) $$A_{\mathrm{dS}}^{\left(0\right)}=\frac{\ell_{\mathrm{Pl}}^{2}}{\pi }\;\gamma \left|\theta^{\left(0\right)}\right|,$$

which, for order-one parameters, is indeed of Planckian magnitude. In the special case $\theta^{\left(0\right)}=\pi$, this reduces to the suggestive form $A_{\mathrm{dS}}^{\left(0\right)}=\gamma \;\ell_{\mathrm{Pl}}^{2}$.

This quantization underscores the central role of the Immirzi parameter. If γ=0, the entropy would be trivialized to zero for the k=0 sector, contradicting the area law given that $\theta^{\left(0\right)}\in \left(0,2\pi \right]$. A nonzero γ is therefore essential for a consistent entropy spectrum.

The degeneracy of states at a fixed *k*-level is defined as

(30) $$N^{\left(k\right)}=e^{S_{\mathrm{dS}}^{\left(k\right)}}=N^{\left(0\right)}e^{\Delta S_{\mathrm{dS}}^{\left(k\right)}},$$

with

(31) $$N^{\left(0\right)}=e^{\gamma |\theta^{\left(0\right)}|/4\pi }.$$

Thus Equation (30) becomes

(32) $$N^{\left(k\right)}=N^{\left(0\right)}e^{\gamma k/2}.$$

Fixing the *k*-level simultaneously fixes the vacuum energy through Equation (14).

The observed value $S_{\mathrm{dS}}∼10^{120}$ is naturally identified with a large topological quantum number *k* labeling our present vacuum sector. This suggests an interpretation of the de Sitter horizon as a topological quantum memory, with each entropy unit corresponding to a topological qubit. The total storage capacity of the Universe is then determined by *k*, with the horizon encoding $k∼10^{120}$ qubits of information.

In this picture, the cosmological constant acquires a direct information-theoretic meaning: it is not merely an energy density but rather a measure of the information density stored on the horizon. A smaller Λ implies a larger horizon area and, correspondingly, a greater quantum memory capacity. The discrete spectrum $\Lambda^{\left(k\right)}∼1/k$ thus implies that each vacuum sector corresponds to a universe with a different information storage capacity, with our observed vacuum being one of enormous information content.

This interpretation is corroborated by the scaling relations

(33) $$|\alpha_{GB}^{\left(k\right)}\left|∼\right|\theta^{\left(k\right)}|∼v^{\left(k\right)}∼S_{\mathrm{dS}}^{\left(k\right)}∼k,$$

which establish a direct correspondence among the Gauss–Bonnet coupling, the gravitational θ-angle, the Higgs VeV, the de Sitter entropy, and the topological index. Conversely, the effective gravitational coupling at the energy scale $\epsilon^{\left(k\right)}$ scales as $|\alpha_{G}^{\left(k\right)}|∼G\Lambda^{\left(k\right)}∼1/k$, suggesting a duality, $|\alpha_{GB}|\;\alpha_{G}∼1$, that remains invariant under any *k*-transformation.

The quantization $S_{\mathrm{dS}}^{\left(k\right)}∼k$ finds a natural interpretation as a four-dimensional Chern–Simons level quantization. In 2+1 dimensions, the de Sitter entropy is $S=2\pi k_{\mathrm{CS}}$, with $k_{\mathrm{CS}}$ the quantized Chern–Simons level [61]. Our result extends this to 3+1 dimensions, identifying $k_{\mathrm{CS}}∼k$, so that the observed entropy $S_{\mathrm{dS}}∼10^{120}$ corresponds to a macroscopic topological charge $k_{\mathrm{CS}}∼10^{120}$. This suggests that the de Sitter horizon is a highly excited state of the pre-geometric Chern–Simons theory.

### 5.2. Thermodynamics of the Quantized Spectrum

The quantization of the vacuum energy also induces a discrete spectrum for the de Sitter temperature. The Gibbons–Hawking temperature of the cosmological horizon is

(34) $$T_{\mathrm{dS}}^{\left(k\right)}=\frac{1}{2\pi }\sqrt{\frac{\Lambda^{\left(k\right)}}{3}}=\frac{\epsilon^{\left(k\right)}}{2\pi },$$

where $\epsilon^{\left(k\right)}=\sqrt{\Lambda^{\left(k\right)}/3}$ corresponds to the Hubble scale of the *k*-th vacuum sector. Substituting the quantized spectrum from Equation (14), we obtain

(35) $$T_{\mathrm{dS}}^{\left(k\right)}=\frac{M_{\mathrm{Pl}}}{\sqrt{\gamma (|\theta^{\left(0\right)}|+2\pi k)}}.$$

This relation suggests a direct connection between the temperature and the information content of the horizon: hotter de Sitter phases correspond to smaller entropy and thus fewer topological qubits. We note that the case γ=0 at k=0 would lead to a divergent temperature, indicating that a nonzero Immirzi parameter is required for consistency. Similarly, $\theta^{\left(0\right)}=0$ is excluded for the k=0 sector to yield a well-defined temperature. These considerations further reinforce the physical necessity of a nontrivial topological sector in the present framework.

## 6. Degeneracy and Emergent N-Portrait

The results presented so far—the quantization of Λ, $S_{\mathrm{dS}}$, and the topological index *k*—follow directly from the meta-geometric construction and the topological periodicity of the θ-angle. We now turn to the interpretation of these results. The identification of *k* with the number of holographic qubits, the number of hairons, and the occupation number *N* of soft gravitons in the *N*-portrait is a suggestive correspondence that motivates further investigation, but it is not derived from first principles within the present framework. These identifications should be regarded as conjectural interpretations that provide a physical picture for the topological quantization, rather than as established results. A microscopic derivation of the area law from the pre-geometric degrees of freedom, which would establish these correspondences rigorously, remains an open challenge.

Moreover, the quantization of the cosmological constant directly implies a *k*-dependent scaling of the tetrads:

(36) $$e_{\mu }^{a(k)}=\frac{1}{\epsilon^{\left(k\right)}}A_{\mu }^{a5},\;\omega_{\mu }^{ab}=A_{\mu }^{ab},$$

with

(37) $$\epsilon^{\left(k\right)}=\sqrt{\frac{\Lambda^{\left(k\right)}}{3}}=\frac{M_{Pl}}{\sqrt{S_{\mathrm{dS}}^{\left(k\right)}/\pi }}.$$

While the tetrads acquire a *k*-dependent rescaling, the spin connection remains *k*-independent. This structure is compatible with the metricity condition

(38) $$\nabla_{\mu }e_{\nu }^{a(k)}+\omega_{\mu \;b}^{\;a}e_{\nu }^{b(k)}=0,$$

where $\nabla_{\mu }$ is the covariant derivative defined with respect to $\omega_{\mu }^{ab}=A_{\mu }^{ab}$. The *k*-factors cancel identically in this equation, leaving no *k*-dependence for the spin connection.

Since tetrads encode the metric structure—and ultimately gravitons upon quantization—the metric tensor can be rewritten as

(39) $$g_{\mu \nu }^{\left(k\right)}=\eta_{ab}e_{\mu }^{a(k)}e_{\nu }^{b(k)}=\frac{\ell_{Pl}^{2}}{\pi }\;S_{\mathrm{dS}}^{\left(k\right)}\;A_{\mu }^{a5}A_{\nu }^{a5},$$

exhibiting a direct proportionality between the metric and the de Sitter entropy, both scaling as *k*. Equivalently, in terms of the Higgs VEV, $g_{\mu \nu }^{\left(k\right)}∼{\langle \Phi \rangle }^{\left(k\right)}A_{\mu }^{a5}A_{\nu }^{a5}$. This suggests that gravitons dissolve in the limit ${\langle \Phi \rangle }^{\left(k\right)}=v^{\left(k\right)}\to 0$, corresponding to the restoration of the gauge symmetry.

The correspondence between the degeneracy of states contributing to the entropy and the graviton degeneracy hints at the emergence of the *N*-portrait picture of cosmology [62,63], from our pre-geometric gauge theory in the Higgsed phase. In the *N*-portrait framework, a macroscopic horizon is understood as a coherent state of *N* soft gravitons, where the entropy scales as S∼N and the coupling weakens as α∼1/N. In our construction, the identification $S_{\mathrm{dS}}^{\left(k\right)}∼k$ naturally maps the topological level *k* to the graviton occupation number *N*, suggesting that the de Sitter horizon corresponds to a highly occupied graviton condensate with $N∼k∼10^{120}$. Moreover, the *k*-dependent rescaling of the tetrads can be interpreted as the manifestation of the backreaction of this condensate on the background geometry, in close analogy with the self-sustained classicalization mechanism of the *N*-portrait. The Higgsed phase of our pre-geometric theory thus provides a microscopic origin for the emergent *N*-portrait structure, wherein the graviton condensate is sourced by the topological Chern–Simons sector and the entropy counts the underlying microstates of the pre-geometric field configuration. Furthermore, this topological interpretation dovetails naturally with the *N*-portrait, as both frameworks equate the entropy with a large integer counting the degrees of freedom of the condensate, thereby offering a unified description of the microscopic quantum structure of the cosmological horizon. A potentially intriguing extension would be to consider black hole solutions as gravitational condensates with de Sitter interiors, where the core and the exterior vacuum are separated by a meta-Higgs shell—possibly carrying a distinct topological index $k^{\prime }$—thereby interpolating between different meta-Higgs vacua across barriers. While this application is suggestive, it requires a significantly more comprehensive analysis and is therefore postponed to future investigations.

We emphasize that the correspondence between the topological index *k* and the graviton occupation number *N* is a conjecture. While it is natural to identify the degeneracy of states $N^{\left(k\right)}∼e^{k}$ with the number of soft gravitons in the *N*-portrait, a rigorous derivation of this identification would require a detailed analysis of the pre-geometric gravitational propagator and its relation to the topological sectors. We leave this for future work.

### 6.1. Quantum Tunneling Transitions and Relaxation Dynamics

We now develop the dynamical framework that describes the quantum evolution of the vacuum energy across the discrete landscape of topological sectors. The central insight is that the total transition rate must incorporate not only the Euclidean bounce action but also the entropy of the final state, which counts the number of available microstates.

For a single transition from sector *k* to sector $k^{\prime }$, the bare quantum tunneling rate into a *specific* final vacuum state is given by the standard instanton result:

(40) $$R_{\mathrm{bare}}^{\left(k\to k^{\prime }\right)}∼e^{-S_{E}^{\left(kk^{\prime }\right)}},$$

where $S_{E}^{\left(kk^{\prime }\right)}$ is the Euclidean bounce action. However, in a landscape with multiple vacua, the *total* rate into *any* state at level $k^{\prime }$ must include the degeneracy factor $N(k^{\prime })$, which counts the number of available final states:

(41) $$R_{k\to k^{\prime }}=N\left(k^{\prime }\right)\;e^{-S_{E}^{\left(kk^{\prime }\right)}}.$$

From the quantization condition $S_{\mathrm{dS}}^{\left(k\right)}∼k$, the de Sitter entropy is quantized. The number of microstates is related to entropy by the Boltzmann relation:

(42) $$N\left(k\right)=e^{S_{\mathrm{dS}}^{\left(k\right)}}∼e^{k}.$$

(For simplicity we set the Immirzi parameter γ to be =1.) Therefore, the complete transition rate becomes

(43) $$R_{k\to k^{\prime }}∼e^{k^{\prime }-S_{E}^{\left(kk^{\prime }\right)}}.$$

Note that the initial sector *k* does not receive an entropy factor N(k), because the coarse-grained probability $P_{k}$ already sums over all microstates in sector *k*. The rate $R_{k\to k^{\prime }}$ is the transition rate from a specific initial microstate (or averaged over initial states with equal weight) to any final microstate in sector $k^{\prime }$. This directly implies that

(44) $$R_{k\to k^{\prime }}\neq R_{k^{\prime }\to k},$$

even in cases where $S_{E}^{\left(kk^{\prime }\right)}=S_{E}^{\left(k^{\prime }k\right)}$—which, in general, is also not true.

The probability distribution $P_{k}\left(\tau \right)$ over the discrete vacuum sectors evolves according to a Markovian Master Equation:

(45) $$\frac{dP_{k}}{d\tau }=\sum_{k^{\prime }\neq k}\left[R_{k^{\prime }\to k}P_{k^{\prime }}-R_{k\to k^{\prime }}P_{k}\right].$$

Here $P_{k}\left(\tau \right)$ denotes the coarse-grained probability that the Universe resides in vacuum sector *k* at time τ, marginalized over the $N\left(k\right)∼e^{k}$ microstates within that sector and satisfying $\sum_{k}P_{k}\left(\tau \right)=1$. Using Equation (43), we obtain

(46) $$\frac{dP_{k}}{d\tau }=\sum_{k^{\prime }\neq k}\left[e^{k-S_{E}^{\left(k^{\prime }k\right)}}P_{k^{\prime }}-e^{k^{\prime }-S_{E}^{\left(kk^{\prime }\right)}}P_{k}\right].$$

The first term represents transitions *into* sector *k* from all other sectors $k^{\prime }$, while the second term represents transitions *out of* sector *k* to all other sectors.

The Master Equation exhibits a fundamental asymmetry. Consider the ratio of transition rates for upward versus downward adjacent transitions:

(47) $$r_{k}=\frac{R_{k\to k+1}}{R_{k\to k-1}}=\frac{e^{\left(k+1\right)-S_{E}^{\left(k,k+1\right)}}}{e^{\left(k-1\right)-S_{E}^{\left(k,k-1\right)}}}.$$

In any case where the entropic factor dominates over the bounce action, this ratio becomes nearly independent of $S_{E}^{\left(k\right)}$, yielding $r_{k}\to e^{\gamma }∼e$. Thus, transitions to higher *k* (smaller cosmological constant) are always entropically favored over transitions to lower *k* by a factor of *e*, purely due to the larger number of available states at higher *k*. For long jumps, the asymmetry is even more pronounced:

(48) $$\frac{R_{k\to k+\Delta N}}{R_{k\to k-\Delta N}}∼e^{\Delta N},\;\left(\gamma ∼1\right).$$

This demonstrates that in the entropically dominant regime—typical for the large-*k* limit—the system exhibits a *net drift* towards larger values of the topological index *k*, corresponding to smaller values of the cosmological constant.

The entropy factor $N\left(k^{\prime }\right)∼e^{S_{\mathrm{dS}}^{\left(k^{\prime }\right)}}$ in Equation (41) arises from summing the bare tunneling rate over all available final microstates in sector $k^{\prime }$. This is a standard Fermi-golden-rule-like counting: the total rate into a degenerate final manifold is the single-channel rate multiplied by the number of available channels. The initial sector *k* does not receive an analogous factor because the coarse-grained probability $P_{k}$ already sums over all microstates in sector *k*. This asymmetry between initial and final state counting is the origin of the effective irreversibility of the Master Equation. At the microscopic level, the underlying quantum gravitational dynamics is assumed to be unitary, and the individual transition amplitudes satisfy detailed balance. The irreversibility emerges only after coarse-graining.

To estimate the Euclidean bounce action, we employ the thick-wall approximation, which is appropriate when the scalar field variation occurs over a scale comparable to its Compton wavelength. The instanton action is then given by

(49) $$S_{E}^{\left(k\right)}∼r_{\rho ,k}^{4}\;\Delta V_{k}/\epsilon ∼\Delta V_{k}/m_{\rho }^{4}\epsilon ,$$

where $r_{\rho ,k}∼m_{\rho (k)}^{-1}$ is the scalar Compton length, proportional to the inverse of the meta-Higgs fluctuation mass $\rho \left(k\right)=\Phi -v_{k}$, and serves as the relevant integration cutoff for the Euclidean integral; $\Delta V_{k}=V_{k+1}-V_{k}$ is the potential difference between adjacent sectors. From the pre-geometric scaling relations, we have

(50) $$\Delta V_{k}∼h_{\mathrm{SSB}}^{\left(k\right)}\epsilon_{k}^{4}v_{k}^{2}\epsilon ∼g_{\mathrm{MM}(k)}^{-2}m_{\rho (k)}^{2}\epsilon .$$

Combining these results yields the bounce action scaling

(51) $$S_{E}^{\left(k\right)}∼g_{\mathrm{MM}(k)}^{-2}m_{\rho (k)}^{-2}.$$

In the large-*k* limit, the thin-wall approximation becomes physically appropriate, where $S_{E}^{\left(k\right)}∼\sigma_{k}r_{k}^{3}$ with $r_{k}∼m_{\rho (k)}^{-1}$. For a tension $\sigma_{k}∼{\left(g_{\mathrm{MM}(k)}^{2}r_{k}\right)}^{-1}$, this yields the holographic scaling $S_{E}^{\left(k\right)}∼g_{\mathrm{MM}(k)}^{-2}r_{k}^{2}∼g_{\mathrm{MM}(k)}^{-2}m_{\rho (k)}^{-2}$, matching the thick-wall result and confirming the robustness of Equation (51).

We now apply this general formalism to the specific scaling predicted by the pre-geometric gravity framework. From the relation $m_{\rho }^{\left(k\right)}∼M_{\mathrm{Pl}}\;k^{-p}$, where $p=c_{\mathrm{ssb}}+c_{\mathrm{MM}}-3$ encodes the scaling of the SSB parameters, and with $g_{\mathrm{MM}}∼M_{\mathrm{Pl}}^{-1}$, the Euclidean action scales as

(52) $$S_{E}^{\left(k\right)}∼g_{\mathrm{MM}}^{-2}m_{\rho }^{-2}∼k^{2p}.$$

The dynamics is governed by the Master Equation (46), and the behavior of the system depends critically on the scaling of $S_{E}^{\left(k\right)}$ with *k*. We can distinguish three regimes.

**(i) Bounce-dominated regime,** p≥1/2: This corresponds to a scalaron mass $m_{\rho }^{\left(k\right)}≲M_{\mathrm{Pl}}/\sqrt{k}$, i.e., at or below the Hubble rate. The WKB approximation is valid when the bounce action is large compared to the de Sitter entropy:

(53) $$S_{E}^{\left(k\right)}≳S_{\mathrm{dS}}^{\left(k\right)}∼k.$$

The most physically motivated case is p=1/2, i.e., $m_{\rho }^{\left(k\right)}∼M_{\mathrm{Pl}}/\sqrt{k}∼\sqrt{\Lambda_{k}}$, corresponding to a scalaron that saturates the Hubble rate. In this case the transition rate is exponentially suppressed as $e^{-k}$, and the Master Equation reduces to

(54) $$\frac{dk}{d\tau }∼A\;e^{-k}\;⟹\;e^{k}dk∼A\;d\tau \;⟹\;k\left(\tau \right)∼\ln\left(A\tau +e^{k_{0}}\right)∼\ln\left(A\tau \right),$$

for $\tau \gg \tau_{\mathrm{Pl}}$ and $A=M_{\mathrm{Pl}}$. This yields the relaxation law

(55) $$\Lambda \left(\tau \right)∼\frac{M_{\mathrm{Pl}}^{2}}{\ln(M_{\mathrm{Pl}}\tau )}.$$

For generic p>1/2, the transition rate is $R∼e^{-S_{E}^{\left(k\right)}+S_{\mathrm{dS}}^{\left(k\right)}}=e^{-k^{2p}+k}≃e^{-k^{2p}}$, so the Master Equation becomes

(56) $$\frac{dk}{d\tau }∼A\;e^{-k^{2p}}\;⟹\;\int^{k}e^{u^{2p}}du∼A\tau .$$

For 2p>1, the integral is dominated by the upper limit, leading to

(57) $$e^{k^{2p}}∼A\tau \;⟹\;k\left(\tau \right)∼{\left(\ln(M_{\mathrm{Pl}}\tau )\right)}^{1/(2p)},$$

and therefore

(58) $$\Lambda \left(\tau \right)∼\frac{M_{\mathrm{Pl}}^{2}}{{\left(\ln(M_{\mathrm{Pl}}\tau )\right)}^{1/(2p)}}.$$

For p=1/2, this reduces to the logarithmic relaxation (55), while for p>1/2 the relaxation is slower, with Λ(τ) decaying as an inverse power of $\ln(M_{\mathrm{Pl}}\tau )$. However, p>1/2 corresponds to ρ-masses below the Hubble rate $H∼M_{\mathrm{Pl}}/\sqrt{k}$, i.e., effectively massless. We discard this possibility, as it would introduce massless dilaton-like new long-range interactions.

**(ii) Critical case,** $S_{\mathrm{dS}}=S_{E}$: When the bounce action and the entropy share the same *k*-scaling, the two factors in the exponential cancel and the relaxation dynamics can be much faster:

(59) $$\mathrm{Critical}\;\mathrm{Case}:\;\Lambda \left(\tau \right)∼\frac{M_{\mathrm{Pl}}}{\tau +\tau_{1}}.$$

Note that the numerical O(1) coefficient multiplying the coupling constant $g_{\mathrm{MM}}$ in $S_{E}$ is a free parameter that determines which contribution dominates—the bounce action or the entropy—when both share the same *k*-scaling.

**Entropically dominated regime,** p<1/2: Here the barrier is negligible compared to the entropic factor, and the evolution is entirely thermal, independent of the quantum tunneling rate. This includes the case p=0, corresponding to a *k*-scale-invariant Euclidean action $S_{E}={\bar{S}}_{E}=\mathrm{const}$ and $m_{\rho }∼M_{\mathrm{Pl}}$. In this regime the Master Equation gives

(60) $$\frac{dk}{d\tau }∼A\;e^{k}\;⟹\;k\left(\tau \right)∼-\ln\left(e^{-k_{0}}-A\tau \right),$$

which diverges at the finite time $\tau_{s}=e^{-k_{0}}/A$. This finite-time singularity signals a breakdown of the effective description, since an infinite tower of higher-order terms would have to be included. Rather than a smooth relaxation, this suggests an uncontrolled cascade toward vacuum energy decay. For $A=M_{\mathrm{Pl}}$ and $k_{0}∼1$, this process would occur on a Planckian time. We postpone a more detailed analysis of this scenario to future work.

In all cases p≥1/2, the system flows toward the asymptotic attractor

(61) $$\lim_{\tau \to \infty }k\left(\tau \right)=\infty \;⟹\;\lim_{\tau \to \infty }\Lambda \left(\tau \right)=0.$$

This represents a universal feature of the p≥1/2 case: $\Lambda_{\infty }=0$ is the unique attractor of the relaxation dynamics. However, the relaxation time diverges as τ→∞ in this limit. The observed small but nonzero value of the cosmological constant may therefore be understood as a reflection of the finite age of the Universe: we occupy the sector $k∼10^{120}$ simply because the Universe has had a finite time to relax from its initial state.

From a microscopic perspective, the increase in *k* corresponds to an increasing number of soft graviton states, which can be understood as an ultra-cold condensate. In the thermodynamic picture, the cosmological constant sets a de Sitter temperature $T∼\sqrt{\Lambda }$; the relaxation dynamics thus corresponds to a spontaneous cooling of the graviton system. This may be interpreted as a splitting dynamics, such as graviton rescatterings 2→m with m>2, which dynamically increase the number of states with lower-energy single partons emerging from the collision center-of-mass energy. In this sense, the arrow of time can emerge microscopically as an overall macroscopic effect of high-multiplicity graviton splitting scatterings. Indeed, in our picture the metric tensor and gravitons are emergent condensates rather than fundamental fields, and as quantum operators they contain an extra quantum number operator compared to the traditional quantum gravity picture. This suggests that graviton creation operators increase with the graviton occupation number, leading to spontaneous multiplicity growth. As shown in Ref. [40], the insertion of such soft gravitons with Standard Model propagating fields can cure UV divergences coming from virtual bubble diagrams, effectively replacing $M_{\mathrm{Pl}}^{2}$ with $\Lambda_{k}$.

### 6.2. Comments on the Inclusion of Matter

A natural question concerns the stability of the meta-Higgs potential against quantum corrections originating from the matter sector, particularly in light of Coleman–Weinberg-type one-loop corrections. This indeed determines not only possible corrections to the cosmological constant but also to the cosmological entropy. The couplings between Standard Model matter and the pre-geometric gravity sector have been systematically addressed in Ref. [1], where it was shown that the standard couplings of the metric tensor to matter fields are recovered in the low-energy limit.

For energies well below the meta-Higgs mass—which is naturally of Planckian order—the fluctuations ρ=Φ−v around the vacuum expectation value are effectively frozen. Consequently, Standard Model corrections to the low-energy propagator of the meta-Higgs field are entirely negligible. Such corrections can only become relevant near the Planck scale, where the theory is expected to enter a non-perturbative regime. Thus, the scale of the meta-Higgs VEV does not experience any significant running at energies far below the Planck scale. Since this VEV controls the bare cosmological constant Λ through the see-saw mechanism discussed above, the resulting contribution to the cosmological constant receives no relevant loop corrections from the meta-Higgs sector.

Moreover, the topological sector of the theory possesses a conserved topological index *k*, which is non-perturbative in nature. Topological invariants of this kind, as well as the associated conductivity constants, are known to be robust against "quantum noise," a feature well established in numerous condensed matter systems such as the quantum Hall effect [64,65,66,67,68,69,70]—see also Ref. [71], where this analogy was first proposed from a different perspective. Similarly, quantum corrections to the potential barriers, which are related to the meta-Higgs mass, are expected to be irrelevant.

The most potentially dangerous contributions to the cosmological constant would appear to arise from vacuum bubble diagrams, which contribute to the energy-momentum tensor as a vacuum energy density and are ultraviolet divergent, thereby threatening to lift the cosmological constant Λ predicted by the meta-Higgs mechanism and the MacDowell–Mansouri construction. However, as previously discussed, several arguments rooted in the holographic naturalness (HN) framework suggest that such contributions are canceled by entropic suppression factors. Moreover, an interplay with the meta-geometric picture can be envisaged, as described in this work: there exists a duality between the large meta-Higgs VEV and the number of soft hairon modes. The number of hairons is a topological index and is therefore exponentially protected against quantum disturbances.

As found in Ref. [1], every Standard Model matter field must couple to the meta-Higgs field through an insertion of its VEV. In the low-energy limit, the coupling of matter to ρ takes the form

(62) $$\left(1+\frac{\rho }{v}+\cdots \right)g_{\mu \nu }T^{\mu \nu },$$

where the ellipsis denotes higher-order coupling terms. This conformal-like coupling implies that, in the dual hairon picture, every matter field is coupled to and entangled with a number of soft hairons scaling as v∼N. Consequently, Standard Model fields experience the vacuum thermal effect, which tames ultraviolet divergences by replacing them with thermal fluctuations proportional to powers of the de Sitter temperature, $∼T_{\mathrm{dS}}^{2p}$. Dually, this can be interpreted as a suppression of every bubble quantum fluctuation as $T_{\mathrm{dS}}^{2p}∼\Lambda^{p}∼{\left(M_{\mathrm{Pl}}/v\right)}^{p}$, with $v\gg M_{\mathrm{Pl}}$—note that this does not imply that the scalar field itself is trans-Planckian, as its magnitude is constrained by the couplings in the potential and gauge sectors. In practice, every matter bubble diagram is replaced by, at minimum, a tadpole diagram with a meta-Higgs vertex, which again amounts to an irrelevant correction to the interaction part of the meta-Higgs potential, polynomially suppressed well below the Planck scale. A full computation and rigorous proof of this mechanism lie beyond the scope of the present work. The arguments presented here should therefore be regarded as suggestive rather than conclusive. In particular, the cancellation of ultraviolet divergences from matter loops through entropic suppression factors remains to be demonstrated explicitly in a concrete model. We emphasize that the claim is not that the cosmological constant problem is solved, but rather that the holographic naturalness framework provides a novel perspective in which the problem is recast in information-theoretic terms.

### 6.3. Finite-Temperature Effects and Symmetry Restoration

An important question concerns the stability of the symmetry-breaking mechanism at finite temperature. The emergence of the geometric phase relies on the spontaneous breaking SO(1,4)→SO(1,3), induced by a nonvanishing vacuum expectation value $\langle \Phi^{A}\rangle =v\delta_{5}^{A}$. At finite temperature, the zero-temperature potential U(|Φ|) is replaced by a thermal effective potential $V_{\mathrm{eff}}\left(\Phi ,T\right)$, which includes thermal corrections of the form

(63) $$m_{\mathrm{eff}}^{2}\left(T\right)=m_{0}^{2}+cT^{2},$$

where *c* depends on the couplings and field content. If c>0, a sufficiently high temperature may restore the symmetry, driving ${\langle \Phi \rangle }_{T}\to 0$ and potentially restoring the full SO(1,4) gauge symmetry.

In the present framework, this possibility is particularly interesting because symmetry restoration would not be a conventional internal-symmetry phenomenon only: since the tetrad emerges from the broken gauge connection, restoration of SO(1,4) would correspond to a transition from the low-temperature geometric phase back to a pre-metric or meta-geometric phase. This could provide a cosmological interpretation of the model, with the ordinary geometric phase emerging through a thermal phase transition as the Universe cools.

The critical temperature $T_{c}$ can be estimated by setting $m_{\mathrm{eff}}^{2}\left(T_{c}\right)=0$, giving $T_{c}∼m_{0}/\sqrt{\left|c\right|}$. For $m_{0}∼M_{\mathrm{Pl}}$, this gives $T_{c}∼M_{\mathrm{Pl}}$, which is of the order of the Planck scale. Thus, symmetry restoration would occur only in the very early Universe, at temperatures comparable to the Planck scale, where the semiclassical description of spacetime is expected to break down anyway. For $T\ll M_{\mathrm{Pl}}$, the symmetry is broken and the geometric phase is stable.

However, this simple estimate assumes that the meta-Higgs mass is of order the Planck scale. If the meta-Higgs is lighter, the critical temperature could be lower, potentially affecting the cosmological evolution of the *k*-sectors. A detailed analysis of the thermal history of the meta-Higgs sector, including the possibility of thermal inflation or a phase transition, is beyond the scope of the present work but would be an interesting direction for future research.

Regarding the *k*-labeled vacuum structure: if thermal corrections modify the positions, depths, or barriers of the minima of the meta-Higgs effective potential, they may also modify the stability of the *k*-vacua and the transition rates between them. In particular, it would be important to understand whether the discrete vacuum structure survives above $T_{c}$ and whether the entropic stabilization of the small-Λ phase remains operative in the early Universe. We leave these questions for future investigation.

## 7. Conclusions and Remarks

In this work, we have shown that a quantized law for the cosmological holographic entropy emerges naturally within a pre-geometric gauge theory of gravity, when combined with the standard semiclassical de Sitter entropy relation. Our construction, based on the MacDowell–Mansouri action augmented by a topological θ-term and a meta-Higgs potential, provides a microscopic origin for the de Sitter entropy as a topological charge of the vacuum.

A central result of our analysis is the quantization of the cosmological constant and the de Sitter entropy according to $\Lambda^{\left(k\right)}∼M_{\mathrm{Pl}}^{2}/k$ and $S_{\mathrm{dS}}^{\left(k\right)}∼k$, where *k* is a discrete topological index. This quantization arises from the periodicity of the gravitational θ-angle and its relation to the Gauss–Bonnet coupling through the Immirzi parameter, uniquely fixed in our framework. The entropy thus acquires a direct information-theoretic interpretation: each unit of *k* corresponds to a topological qubit stored on the cosmological horizon, with the observed value $S_{\mathrm{dS}}∼10^{120}$ reflecting a macroscopic topological charge.

The correspondence between the entropy and the topological index naturally maps onto the *N*-portrait picture of cosmology, wherein the de Sitter horizon is interpreted as a coherent state of N∼k soft gravitons. The *k*-dependent rescaling of the tetrads, while leaving the spin connection invariant, provides a geometric manifestation of the graviton condensate backreaction, offering a unified description of the microscopic quantum structure of the cosmological horizon.

We have further developed a dynamical framework describing the quantum evolution of the vacuum energy across the discrete landscape of topological sectors. The Master Equation governing the probability distribution over the discrete vacua exhibits a fundamental asymmetry: transitions to higher topological index (smaller cosmological constant) are entropically favored, purely due to the larger number of available microstates. The behavior of the system depends critically on the scaling of the Euclidean bounce action with the topological index, parametrized by the exponent *p* of the scalaron mass. We have identified three regimes: (i) the bounce-dominated case p≥1/2, which includes the physically motivated case p=1/2 with a scalaron mass $m_{\rho }∼M_{\mathrm{Pl}}/\sqrt{k}$ saturating the Hubble rate, yielding a logarithmic relaxation $\Lambda \left(\tau \right)∼M_{\mathrm{Pl}}^{2}/\ln\left(M_{\mathrm{Pl}}\tau \right)$, and the more general p>1/2 case with slower, inverse-power-of-log relaxation; (ii) a critical case where the bounce action and the de Sitter entropy share the same scaling, yielding $\Lambda \left(\tau \right)∼M_{\mathrm{Pl}}/\left(\tau +\tau_{1}\right)$; and (iii) the entropically dominated regime p<1/2, which leads to a finite-time singularity and an uncontrolled cascade toward vacuum energy decay. In all cases p≥1/2, the system flows toward the universal attractor $\Lambda_{\infty }=0$, with the observed small but nonzero value of the cosmological constant reflecting the finite age of the Universe.

Our results offer a concrete realization of the holographic naturalness program: the cosmological constant is stabilized not by symmetry or fine-tuning, but by the immense entropic barrier $e^{-S_{\mathrm{dS}}}$ that suppresses transitions to high-energy vacua. The stability of the observed vacuum state is therefore a consequence of statistical typicality and information-theoretic suppression, rather than energetic minimization.

Finally, we have commented on finite-temperature effects and the possibility of symmetry restoration, noting that the geometric phase is expected to be stable for $T\ll M_{\mathrm{Pl}}$, but a detailed analysis of the thermal history of the meta-Higgs sector remains an open problem.

It is worth noting that the relaxation of the cosmological constant described in this work can be related to the effective description of cosmic acceleration in terms of a viscous cosmic fluid. In viscous fluid cosmology, the cosmic acceleration can be driven not only by a bare cosmological constant but also by bulk viscous stresses in the cosmic fluid. The effective pressure acquires a correction of the form Π=−3ξH, where ξ is the bulk viscosity coefficient and *H* is the Hubble parameter. This leads to an effective negative pressure and can drive accelerated expansion even in the absence of a cosmological constant. In our framework, the time-dependent relaxation of Λ(τ) toward smaller values provides a microscopic origin for a dynamical dark-energy component, which can be mapped onto an effective viscous fluid description. The correspondence is not exact, but it suggests that the pre-geometric relaxation mechanism may be phenomenologically indistinguishable from a viscous dark-energy model at late times, at least at the level of the background expansion. The foundational works on causal relativistic viscous fluid dynamics [72,73,74] provide the theoretical framework, while the application of bulk viscosity to cosmic acceleration and dark energy has been extensively studied in the literature [75,76,77,78,79,80,81,82,83,84,85,86]. For a recent comprehensive review, see Ref. [87].

## Data Availability

The original contributions presented in this study are included in the article. Further inquiries can be directed to the corresponding author.
